# Spectral Sharpening of Color Sensors: Diagonal Color Constancy and Beyond

**DOI:** 10.3390/s140303965

**Published:** 2014-02-26

**Authors:** Javier Vazquez-Corral, Marcelo Bertalmío

**Affiliations:** Information and Communications Technologies Department, Universitat Pompeu Fabra, Roc Boronat 138, Barcelona 08018, Spain; marcelo.bertalmio@upf.edu

**Keywords:** spectral sharpening, computational color constancy, color sensors

## Abstract

It has now been 20 years since the seminal work by Finlayson *et al.* on the use of spectral sharpening of sensors to achieve diagonal color constancy. Spectral sharpening is still used today by numerous researchers for different goals unrelated to the original goal of diagonal color constancy e.g., multispectral processing, shadow removal, location of unique hues. This paper reviews the idea of spectral sharpening through the lens of what is known today in color constancy, describes the different methods used for obtaining a set of sharpening sensors and presents an overview of the many different uses that have been found for spectral sharpening over the years.

## 1. Introduction

Our visual system has a striking ability in allowing us to deal with color. However, we are far from fully understanding its behavior. To gain an insight into how our visual system works, some assumptions are often made: first, it is assumed that there is a single illuminant in the scene which is spatially uniform, and second, it is assumed that objects are flat, coplanar, and Lambertian, i.e., their reflectances are diffuse and independent from the angle of view.

Following these assumptions light energy reaching our eye depends on the spectral power distribution of the illuminant (*E*(λ), where λ spans the visible spectrum) and the spectral reflectance distribution of the object we are looking at (*R*(λ)). This information is called the color signal and is written as

(1) C(λ)=R(λ)E(λ)

This color signal is weighted along the visible spectrum *ω* with the sensitivities of our cone-cells (which peak in the long, medium and short wavelengths of the visible range, and are denoted by *s*(λ),*m*(λ),*l*(λ) respectively) to obtain the L,M,S color space coordinates of the signal

(2) $$\{L,M,S\}=\int_{\omega }C(\lambda )\{l,m,s\}(\lambda )d\lambda$$

From this equation, we can see that when looking at a white piece of paper in sunset the values captured by our eye are reddish as a result of the illumination. In contrast, when looking at a white piece of paper on a cloudy day these values are bluish. However, we perceive the piece of paper as approximately white in both cases. This property of our visual system is called color constancy.

### 1.1. Human Color Constancy

**Figure 1.**
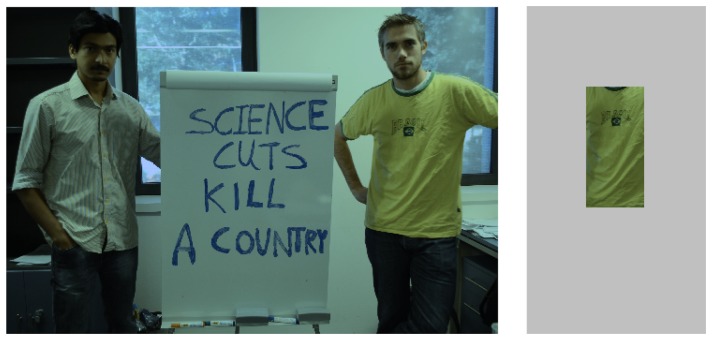
Example of color constancy. We are able to perceive the t-shirt of the man in the right as yellow, but, when looking at it in isolation the color of the t-shirt appears green.

Color constancy is usually defined as the effect whereby the perceived or apparent color of a surface remains constant despite changes in intensity and spectral composition of the illumination [1]. An example is shown in Figure 1, where we are able to perceive the t-shirt of the man in the right as yellow; however, if we isolate the t-shirt we perceive it as green. Some reviews on color constancy have been published recently [1–3].

von Kries [4] in the XIXth century hypothesized that a compensation (or gain) normalization was performed individually within each photoreceptor. Mathematically, we may write

(3) $${\left(\begin{array}{c} L\\ M\\ S \end{array}\right)}_{\text{adapted}}=\left(\begin{array}{ccc} g_{1}(L) & 0 & 0\\ 0 & g_{2}(M) & 0\\ 0 & 0 & g_{3}(S) \end{array}\right)\cdot {\left(\begin{array}{c} L\\ M\\ S \end{array}\right)}_{in}$$

where the subscript *in* represents the values captured by the eye.

This model is called von Kries adaptation. The idea of an individual gain for each photoreceptor was adopted early in computer vision and the gains were computed based on the scene, e.g., assuming that the scene had a mean value of grey [5], or that a white-patch was presented on the scene [6]. von Kries adaptation has been shown to provide a reasonable approximation of how we perceive scenes composed of natural reflectances and illuminant spectra [3]. In a contemporany version of the model, it is stated that cone-signals interact and the gain in each channel is influenced by the other channels [7]

(4) $${\left(\begin{array}{c} L\\ M\\ S \end{array}\right)}_{\text{adapted}}=\left(\begin{array}{ccc} g_{1}(L,M,S) & 0 & 0\\ 0 & g_{2}(L,M,S) & 0\\ 0 & 0 & g_{3}(L,M,S) \end{array}\right)\cdot {\left(\begin{array}{c} L\\ M\\ S \end{array}\right)}_{in}$$

Even though this model can predict the data very well in natural environments, there is still no agreement in the literature as to how the gain values are computed from the image statistics of the stimulus [3]. Furthermore, this model performs poorly when natural reflectances and illuminants are not present in the stimulus.

For this reason, further research on the neural mechanisms that underline color constancy has been conducted over the years [1]. Different parts of the brain have been shown to deal with color constancy, specially the lateral geniculate nucleus (LGN), and the regions V1 and V4 of the visual cortex [1], although recent studies suggest that other areas might also be involved [8]. Therefore, as Smithson says [2] “*It seems most reasonable to say that processing for color constancy starts in the retina*, *is enhanced in V1*/*V2 and continues in V4*”. Cues for color constancy used by humans that might befall in the further neural levels might include mutual reflections, 3D shapes, shadows, color memory, and even the consciousness of illumination change [2,9].

### 1.2. Computational Color Constancy

Computational color constancy does not aim to recover the perceived image, but an estimation of the surface reflectances of the scene; Therefore, it changes the paradigm upon which human color constancy is built. In other words, while human color constancy relies on the perception of the colors, computational color constancy relies on the absolute color values of the objects viewed under a canonical (usually white) illuminant, without considering how the image is perceived by an observer. Vazquez-Corral *et al.* [10] have shown that computational and human color constancy do not aim for the same final image: they performed a pair-wise comparison experiment where they asked human observes to pick out the most natural image from a range of images produced by different computational color constancy algorithms. Observers only chose the best computational solution in 40% of the comparisons.

Therefore, computational color constancy is treated from a mathematical point of view. Let us suppose we have an object with reflectance *R*(λ) and a camera with sensitivities *Q_i_*(λ). We take two photos of the object with the camera at two different moments in time; at each of these moments there is a different illuminant in the scene *E*^1^(λ), *E*^2^(λ) (let us suppose the illuminant is uniform). Then, the response of the object recorded by the camera sensor *i* under one of the illuminants is denoted by $\rho_{i}^{n}$ where the superscript *n* denotes the illuminant used. Mathematically,

(5) $$\begin{array}{ll} \rho_{i}^{1} & =\int R(\lambda )E^{1}(\lambda )Q_{i}(\lambda )d\lambda \\ \rho_{i}^{2} & =\int R(\lambda )E^{2}(\lambda )Q_{i}(\lambda )d\lambda \end{array}$$

Marimont and Wandell [11] showed that although natural surface reflectances in the world are 5 or 6 dimensional, i.e., there is a need of a basis of 5 or 6 reflectances in order to derive any other reflectance, an “effective basis” of smaller dimension can be extracted. Similarly, Judd *et al.* [12] showed how to derive a basis from the set of daylight illuminations. These studies allow us to relate $\underline{\rho^{1}}=[\rho_{1}^{1},\rho_{2}^{1},\rho_{3}^{1}]$ and $\underline{\rho^{2}}=[\rho_{1}^{2},\rho_{2}^{2},\rho_{3}^{2}]$ by a 3 × 3 matrix. That means, we are dealing with a 9-dimensional problem:

(6) $${\underline{\rho }}^{1}=M^{1,2}{\underline{\rho }}^{2}.$$

Equation (6) is crucial in order to solve the computational color constancy problem. If we wish to estimate reflectances by discounting the color of the prevailing light from the picture of a scene, it suffices to find the matrix that replaces this light by the canonical one. As an example, if an image is captured under bluish light then all the recorded sensor responses are biased in the blue direction and, in particular, a white surface will itself be bluish. If we can find the matrix that takes us from the blue light to a white counterpart then applying this matrix will remove the colored light. Of course, as simple as Equation (6) is, there are 9 components in a 3 × 3 matrix and so color constancy, viewed in this perspective, is a hard 9-dimensional problem.

## 2. Spectral Sharpening for Diagonal Color Constancy

Early research in computational color constancy [5,6] focused on modeling illumination changes by a scaling multiplication in each of the sensor channels (inspired by von Kries' coefficient law). This idea, called the diagonal model of illuminant change, can be expressed as:

(7) $${\underline{\rho }}^{1}\approx D^{1,2}{\underline{\rho }}^{2}$$

where *D*^1,2^ is a 3 × 3 diagonal matrix. Equation (7) supposes that illumination change is a process that operates in each sensor channel independently, which simplifies color constancy computation. The diagonal model turns out to be rather good at accounting for illuminant change in many circumstances. Partly, this is explained by the underlying physics which states that as the support of the sensor becomes small, i.e., as the range of wavelengths to which the sensor responds becomes smaller, then a diagonal matrix will work well [13]. Empirically, it has been shown that a diagonal matrix works for most cameras that have spectral sensitivities with support of 100 to 150 nanometers [14]. Let us in any case note that this is different from von Kries adaptation, which should be performed in the L,M,S cone space.

Many cameras do not have sensors that match the above specifications. Therefore, different methods have tried to search for a linear combination of the original sensor responses in order to force them to accomplish the diagonal model. Mathematically, this linear combination will be the one accomplishing

(8) $$T{\underline{\rho }}^{1}\approx D^{1,2}T{\underline{\rho }}^{2}$$

**Figure 2.**
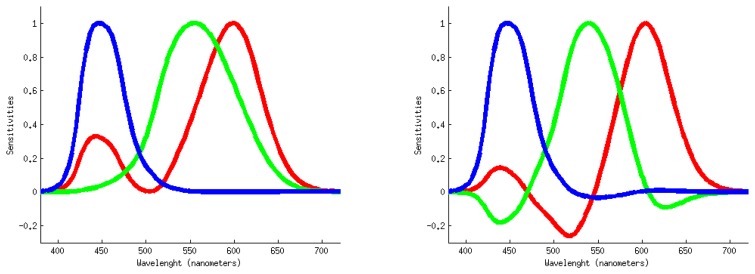
Original camera sensors (**left**) and their sharpened counterparts (**right**).

Finlayson *et al.* [15] called this approach “spectral sharpening” since the new sensors responses are sharper than the original ones. We should note that other authors had previously suggested a similar idea [16]. Figure 2 compares a set of originals sensors with their sharpened version. The remarkable and useful conclusion of the spectral sharpening work was that, even for a broad-band sensor system, a diagonal matrix model of illumination could be used to solve the computational color constancy problem.

**Figure 3.**
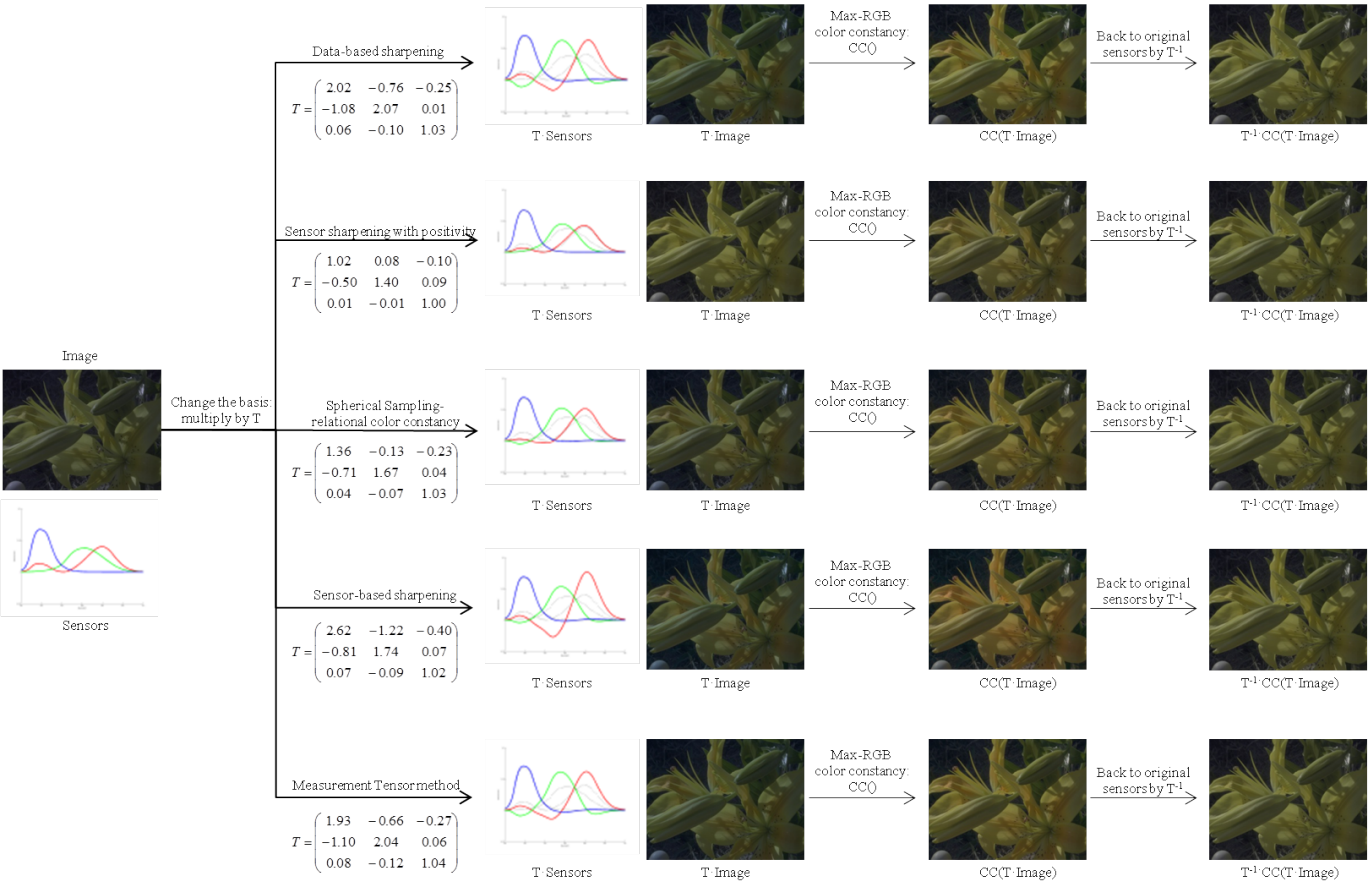
Example of diagonal color constancy using spectral sharpening for five different methods. The original image (sensors) is converted by a linear matrix *T* to a sharpened basis. Then, a diagonal color constancy method is applied (MaxRGB in this case). Finally, the resultant image is converted back to the original basis by the inverse of the matrix *T*. In this example, the original sensors are the CMF XYZ functions. The sharpening matrices have been obtained using the Planckian illuminants and the whole set of reflectances from [17]. The multispectral image comes from [18].

An example of the use of spectral sharpening can be seen in Figure 3. In this figure, an original image that presents a blue cast is seen under the CMF color matching functions. In order to apply diagonal color constancy and remove the blue cast, a linear transform *T* is computed by 5 different methods (that will be explained later in this section). Then, for each method, the pipeline works as follows: (1) a change of basis is performed by the linear transform *T*; (2) a method for diagonal color constancy (MaxRGB in this case) is applied to the new basis image; (3) the image is converted back to the original sensors by *T*^−1^. The different *T* matrices have been obtained using the Planckian illuminants and the whole set of reflectances from [17].

**Figure 4.**
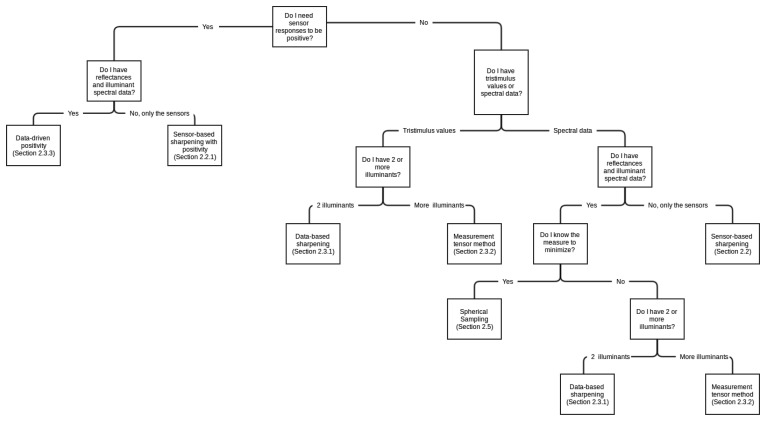
Hierarchy for the selection of a spectral sharpening method. The decision should take into account two aspects: the final goal pursued and the availability of spectral data.

In this section we will show a review of different methods used to achieve spectral sharpening. Figure 4 presents a hierarchy regarding when each particular method might be used. In this figure, the different methods are linked to their section in the paper. The selection of a particular method might be taken depending on two aspects: the availability of spectral data and the final goal that is being pursued.

### 2.1. Perfect Sharpening

Finlayson *et al.* [15,19] showed that when illuminants are two dimensional (in the sense that two illuminants are enough to define any other illuminant as a linear combination of them) and reflectances three dimensional (in the same sense), or vice versa, spectral sharpening is perfect.

Let us suppose that reflectances are three dimensional and illuminants two dimensional (the other case is analogous). In this case any reflectance can be decomposed as

(9) $$R(\lambda )=\sum_{j=1}^{3}R_{j}(\lambda )\sigma_{j}$$

where 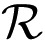
*_j_*(λ) is a basis and *σ̱* = [*σ*_1_, *σ*_2_, *σ*_3_] is a coefficient vector in this basis. Let us define Λ*^k^* as a 3 × 3 matrix which *ijth* entry is defined as $\Lambda_{ij}^{k}=\int_{\omega }Q_{i}(\lambda )E^{k}(\lambda )R_{j}(\lambda )$, where superscript *k* denotes the illuminant used and *Q_i_* are the sensors to be sharpened. Then, a color descriptor (Equation (5)) under a canonical light *c* can be written as

(10) $${\underline{p}}^{c}=\Lambda^{c}\underline{\sigma }$$

As illuminants are two dimensional they need a second illuminant *E*^2^(λ) independent from the canonical one *E^c^*(λ) to span the space. Associated with this illuminant there will also be a new lighting matrix Λ^2^. This second lighting matrix is some linear transform (*M*) away from the first one, Λ^2^ = *M*Λ*^c^*, that is, *M* = Λ^2^[Λ*^c^*]^−1^.

As *E*^2^(λ) and *E^c^*(λ) span the space, any other lighting matrix will be a combination of Λ*^c^* and *M*Λ*^c^*. For this reason, any color descriptor under an illuminant *E^e^*(λ) = *αE^c^*(λ) + *βE*^2^(λ) can be written as

(11) $${\underline{p}}^{e}=[\alpha I+\beta M]\Lambda^{c}\underline{\sigma }=[\alpha I+\beta M]{\underline{p}}^{c}$$

where *I* is the identity matrix. Calculating the eigenvector decomposition of *M*

(12) $$M=T^{-1}DT$$

and expressing the identity matrix in terms of *T*,*I* = *T*^−1^*IT*, they rewrite Equation (11) as a diagonal transform

(13) $$T{\underline{p}}^{e}=[\alpha I+\beta D]T{\underline{p}}^{c}.$$

Finally, writing *p̱^c^* in terms of *p̱^e^*

(14) $$T{\underline{p}}^{c}={\left[\alpha I+\beta D\right]}^{-1}T{\underline{p}}^{e}.$$

This implies that the spectral sharpening is perfect since both matrices *D* and *I* are diagonal.

### 2.2. Sensor-Based Sharpening

Finlayson *et al.* proposed in [15] a method called Sensor-based spectral sharpening. The idea underlying this method is that it is possible to sharpen a sensor from an original set *Q*(λ) (of dimension *n* × *k*) in a wavelength interval [λ_1_, λ_2_]. The resulting sensor *Q*(λ)*ṯ* where *ṯ* is a coefficient vector of dimension *k*, can be found by minimizing

(15) $$\min\sum_{\Phi }{\left[Q(\lambda )\underline{t}\right]}^{2}+\mu \{\int_{\omega }{\left[Q(\lambda )\underline{t}\right]}^{2}d\lambda -1\}$$

where *ω* is the visible spectrum, Φ denotes wavelengths outside [λ_1_, λ_2_] and *μ* is a Lagrange multiplier. In other words, the idea is to strengthen the percentage of the norm of sensor *Q*(λ)*ṯ* lying in the interval [λ_1_, λ_2_] in relation to the rest of the spectrum.

To solve the problem for all the spectra Finlayson and co-authors defined *k* intervals, where *k* is the number of sensors. These intervals have no intersection between them and cover all the spectra. Then, the *kth* row of matrix *T* will be the vector that minimizes Equation (15) for its particular interval (note that *ṯ* is post-multiplying in Equation (15) while *T* is defined in Equation (8) as a pre-multiplication).

Mathematically, they defined a *k* × *k* matrix

(16) $$\Lambda (\alpha )=\sum_{\lambda \in \alpha }Q^{t}(\lambda )Q(\lambda )=Q^{t}(\lambda )\Delta_{\alpha }Q(\lambda )$$

where Δ*_α_* is an operator that picks out wavelengths indices in the sharpening interval *α* within any sum.

They took partial derivatives over the vector *ṯ* in Equation (15) and equated to the zero vector to look for the stationary values. They combined this derivative with Λ(*α*) obtaining:

(17) $$\Lambda (\Phi )\underline{t}+\mu \Lambda (\omega )\underline{t}=0.$$

In parallel, they differentiated Equation (15) over *μ* and found the constraint Σ_λ∈_*_ω_*[*Q*(λ)*ṯ*]^2^ = 1. Rearranging Equation (17), they concluded that finding *ṯ* amounts to solving eigenvector problem

(18) $$\Lambda {\left(\omega \right)}^{-1}\Lambda (\Phi )\underline{t}=-\mu \underline{t}.$$

Then, as this last equation has multiple solutions, they choose one solution minimizing Σ_λ∈Φ_[*Q*(λ)*ṯ*]^2^.

#### 2.2.1. Sharpening with Positivity

Sensors with negative values are physically impossible. For this reason Pearson and Yule [20] defined different positive combinations of the color matching functions. Following this trend, Drew and Finlayson proposed methods to obtain sharpening transforms giving always positive values [21]. These techniques are very similar to the previous one but some constraints were added for ensuring all the values are positive. These constraints can be based either on the *L*_1_ or *L*_2_ norm and can be performed both in the sensors themselves or in the sharpening matrix coefficients. All these methods can be solved by either linear or quadratic programming. Here we report the different methods presented.

- *L*_1_- *L*_1_ Constrained coefficients:
  (19) $$\arg\underset{\underline{t}}{\min}\sum_{\Phi }[Q(\lambda )\underline{t}]\;\text{constrained to}\;\{\begin{array}{c} \min\sum_{\omega }[Q(\lambda )\underline{t}]=1\\ \underline{t}\geq 0 \end{array}$$
- *L*_1_- *L*_1_ Constrained sensors:
  (20) $$\arg\underset{\underline{t}}{\min}\sum_{\Phi }[Q(\lambda )\underline{t}]\;\text{constrained to}\;\{\begin{array}{c} \min\sum_{\omega }[Q(\lambda )\underline{t}]=1\\ Q(\lambda )\underline{t}\geq 0 \end{array}$$
- *L*_2_- *L*_2_ Constrained coefficients:
  (21) $$\arg\underset{\underline{t}}{\min}\sum_{\Phi }{\left[Q(\lambda )\underline{t}\right]}^{2}\text{constrained to}\;\{\begin{array}{c} \min\sum_{\omega }{\left[Q(\lambda )\underline{t}\right]}^{2}=1\\ \underline{t}\geq 0 \end{array}$$
- *L*_2_*- L*_2_ Constrained sensors
  (22) $$\arg\underset{\underline{t}}{\min}\sum_{\Phi }{\left[Q(\lambda )\underline{t}\right]}^{2}\text{constrained to}\;\{\begin{array}{c} \min\sum_{\omega }{\left[Q(\lambda )\underline{t}\right]}^{2}=1\\ Q(\lambda )\underline{t}\geq 0 \end{array}$$
- *L*_2_*- L*_1_ Constrained coefficients
  (23) $$\arg\underset{\underline{t}}{\min}\sum_{\Phi }{\left[Q(\lambda )\underline{t}\right]}^{2}\text{constrained to}\;\{\begin{array}{c} \min\sum_{\omega }\left[Q(\lambda )\underline{t}\right]=1\\ \underline{t}\geq 0 \end{array}$$
- *L*_2_- *L*_1_ Constrained sensors
  (24) $$\arg\underset{\underline{t}}{\min}\sum_{\Phi }{\left[Q(\lambda )\underline{t}\right]}^{2}\;\text{constrained to}\;\{\begin{array}{c} \min\sum_{\omega }\left[Q(\lambda )\underline{t}\right]=1\\ Q(\lambda )\underline{t}\geq 0 \end{array}$$

### 2.3. Adding Information to Improve Sharpening

Information about the illuminants and reflectances that are more representative in natural scenes is available from multiple sources [12,17,22–26]. In this section we review methods that take advantage of this available information to search for sharpened sensors.

#### 2.3.1. Data-Based Sharpening

Finlayson *et al.* [15] proposed a method called data-based sharpening which uses linear algebra methods to directly solve for *T* by minimizing the residual error between a pair of illuminants. To this end, they defined *W*^1^ and *W*^2^ as 3 × *n* matrices containing the color values for a set on *n* different refectances under two different illuminants *E*^1^ and *E*^2^.

(25) $$TW^{1}\approx D^{1,2}TW^{2}.$$

Then, they solved Equation (25) for *D*^1,2^ in a least-squares sense. This can be done by the Moore-Penrose inverse

(26) $$D^{1,2}=TW^{1}{\left[TW^{2}\right]}^{+}$$

where []^+^ represents the pseudoinverse [27]. Rearranging Equation (26), they got

(27) $$T^{-1}D^{1,2}T=W^{1}{\left[W^{2}\right]}^{+}.$$

Therefore, *T* is the eigenvector decomposition of *W*^1^[*W*^2^]^+^.

Some years later, Barnard *et al.* [28] tried to allow more flexibility to the database sharpening, by averaging over a set of illuminants (not only one), and introducing a parameter to prioritize positivity.

#### 2.3.2. Measurement Tensor

Chong *et al.* [29] introduced a new method which finds a matrix *T* for a complete set of illuminants at the same time. This method is based on the measurement tensor defined as

(28) $$M_{\text{kij}}:=\int Q_{k}(\lambda )E_{i}(\lambda )R_{j}(\lambda )d\lambda$$

where {*E_i_*}*_i_*_=1,⋯,_*_I_* is a set of illuminants, {*R_j_*}*_j_*_=1,⋯,_*_J_* is a set of reflectances and {*Q_k_*}*_k_*_=1,⋯,_*_K_* are sensors. This measurement tensor is an order 3 tensor.

Chong *et al.* proved that a measurement tensor supports diagonal color constancy if and only if it is a rank 3 tensor. An order 3 tensor *τ* is rank *N* if *N* is the smallest integer such that there exist vectors ${\left\{\underline{a_{n}},\underline{b_{n}},\underline{c_{n}}\right\}}_{n=1,\cdots ,N}$ allowing decomposition as the sum of outer products

(29) $$\tau =\sum_{n=1}^{N}\underline{c_{n}}\circ \underline{a_{n}}\circ \underline{b_{n}}$$

where ○ represents the outer product.

They rewrote Equation (29) as

(30) $$\tau =\sum_{n=1}^{N}C\circ A\circ B$$

Where the columns of *A*, *B* and *C* are composed by the different $\underline{a_{n}}$, $\underline{b_{n}}$, and $\underline{c_{n}}$ respectively. *C* is the matrix we search, and *T* = *C*^−1^.

In order to solve Equation (30) they used the Trilinear Alternate Least Squares (TALS) method [30]. This is necessary since in most of the cases the tensor *M_kij_* is not rank 3, and therefore it is necessary to search for the “closest” rank 3 tensor. At each iteration of the minimization procedure through TALS, two of the three matrices are fixed while the free matrix is chosen to minimize the difference between the given data *M_kij_* and the obtained tensor *τ* in the least-squares sense. The alternating process is repeated until convergence.

This method has the drawback of local convergence, that is, the result obtained can be a local minima. Also, TALS needs initialization values for two of their three matrices.

#### 2.3.3. Data-Driven Positivity

In [21], Drew and Finlayson proposed a data-driven approach for obtaining positive sensors. Following the original sensor-based sharpening method they divided the spectra in *k* intervals, where *k* is the number of sensors, and for each interval they searched for the sensor *Q*(λ)*ṯ* minimizing

(31) $$\min\sum_{\Phi }{\left[Q(\lambda )\underline{t}\right]}^{\upsilon }\;\text{constrained to}\;\{\begin{array}{c} \min\sum_{\omega }{\left[Q(\lambda )\underline{t}\right]}^{\upsilon }=1\\ \hat{R}\underline{t}\geq 0 \end{array}$$

where Φ denotes the wavelengths outside the selected interval, *ω* represents the visible interval, *R̂* is a *r* × 3 matrix representing the gamut boundary of the set of RGBs obtained from the data, *r* represents the number of points lying on that boundary, and *&upsilon;* = 1, 2 represents the chosen norm. The transpose of the vector *ṯ* is the *k*-th row of the sharpening matrix *T*. This method does not guarantee *per se* the positiveness of the result. Positiveness is conditioned to select a big enough space of reflectances and illuminants so no other color signal lies out of the gamut defined by *R̂*.

### 2.4. Chromatic Adaptation Transforms

All the previous methods were defined in order to help solving for diagonal color constancy. But, the sharpening matrices related to these methods are not the only ones, there are sharpening matrices that have been derived from psychophysical experiments in chromatic adaptation. Chromatic adaptation matrices also represent sharp sensors [31], and are obtained from the XYZ color matching functions. They are used to match image appearance to colorimetry when the visual conditions are changed. In particular, they are defined to handle corresponding colors data. Citing from the Fairchild's book [32] “*Corresponding colors are defined as two stimuli*, *viewed under different viewing conditions*, *that match in color appearance*”. Examples of chromatic adaptation transforms are the Bradford transform, the Fairchild transform, and the CAT02 transform. For a review on the different transforms, we recommend the book by Fairchild [32]. Here we enumerate some of them.

#### 2.4.1. Von Kries Transform

The Von Kries chromatic adaptation transform is usually defined by the Hunt, Poynton and Estevez transform [32]. The values of this transform are:

(32) $$T=\left(\begin{array}{ccc} 0.3897 & 0.6890 & -0.0787\\ -0.2298 & 1.1834 & 0.0464\\ 0 & 0 & 1 \end{array}\right)$$

#### 2.4.2. Bradford Transform

The Bradform transform was defined by Lam [33] following the results obtained in an experiment regarding corresponding colors. The data used for the experiment consisted of 58 dyed wood samples under the *A* and *D*65 illuminants. The original Bradford transform is non-linear, but the non-linear part is usually neglected. The linear matrix is then

(33) $$T=\left(\begin{array}{ccc} 0.8951 & 0.2664 & -0.1614\\ -0.7502 & 1.7135 & 0.0367\\ 0.0389 & -0.0685 & 1.0296 \end{array}\right)$$

#### 2.4.3. Fairchild Transform

The Fairchild transform was suggested by Mark Fairchild [34] for improving the CIECAM97s color appearance model. It was obtained through a linearization of the previous chromatic adaptation transform. The matrix suggested by Fairchild was

(34) $$T=\left(\begin{array}{ccc} 0.8562 & 0.3372 & -0.1934\\ -0.8360 & 1.8327 & 0.0033\\ 0.0357 & -0.0469 & 1.0112 \end{array}\right)$$

#### 2.4.4. CAT02 Transform

The *Comission Internationale de l'Éclairage* (CIE) selected in its report CIC-TC8-01 the CAT02 transform as the preferred chromatic adaptation transform. CAT02 was obtained by optimizing a wide variety of corresponding data, while approximating the non-linear transformation of CIECAM97s. The matrix obtained was

(35) $$T=\left(\begin{array}{ccc} 0.7328 & 0.4296 & -0.1624\\ -0.7036 & 1.6975 & 0.0061\\ 0.0030 & 0.0136 & 0.9834 \end{array}\right)$$

#### 2.4.5. Chromatic Adaptation Transforms by Numerical Optimization

Bianco and Schettini [35] defined two new chromatic adaptation transforms based on estimating the corresponding colors data from [4,33,36–40].

They defined an objective function with two competing terms

(36) $$f_{BS}(T)=g_{\text{est}}(T)-g_{\text{med}}(T).$$

The term *g_est_* becomes bigger for better estimations of corresponding colors according to the Wilcoxon signed-rank test. On the other hand, the term *g_med_* becomes smaller when the median errors on the corresponding colors datasets are smaller. Therefore, our goal must be to look for the transformation *T* maximizing Equation (36). To this end, authors applied the Particle Swarm Optimization (PSO) technique.

This first optimization might, however, incur in negative values of the resulting sensors. For this reason they defined another objective function

(37) $$f_{BS-PC}(T)=g_{\text{est}}(T)-g_{\text{med}}(T)+g_{PC}(T).$$

In this second function they added a positive competing term *g_PC_* that prioritizes positivity of the sensors. The second transform was obtained by maximizing Equation (37) though PSO.

### 2.5. Spherical Sampling

Spherical sampling [31,41] provides a means for discretely sampling points on a sphere and relate them to sensors. The main idea is to consider each row of the sharpening matrix *T* as a point in the sphere.

Mathematically, let us represent our original camera sensors *Q* as a *m* × 3 matrix where *m* is the wavelength sampling and 3 the number of sensors. We perform the reduced singular value decomposition (SVD) of these sensors in order to obtain a basis:

(38) $$Q=U\cdot \sum \cdot V^{t}$$

where *U* is an orthogonal matrix with dimension *m* × 3, Σ is a diagonal 3 × 3 matrix containing the singular values of matrix *Q* and *V^t^* is an orthogonal 3 × 3 matrix. Then, *U* is the basis we seek.

From this basis *U*, we can define a new set of sensors 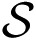 (*m* × 3), different from the original sensors *S*, by multiplying the basis by any linear transformation *P* (3 × 3), which simply consists of 3 sample points vectors, $\underline{p_{1}}$, …, $\underline{p_{3}}$ located over the 2-sphere. Then,

(39) $$\begin{array}{ll} Q=UP, & P=[\underline{p_{1}},\cdots ,\underline{p_{n}}] \end{array}$$

We are interested in the relation between the original sensors *Q* and the new defined ones 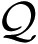. Using Equations (38) and (39) we find

(40) $$Q=UP=U\sum V^{t}{\left(\sum V^{t}\right)}^{-1}P=Q{\left(\sum V^{t}\right)}^{-1}P$$

Therefore, relating this equation to Equation (8) where *T* is pre-multiplying we obtain

(41) $$T={\left({\left(\sum V^{t}\right)}^{-1}P\right)}^{t}$$

We can also rearrange this equation in order to relate a transformation matrix *T* with a set of points *P* over the sphere.

(42) $$P=\sum V^{t}T^{t}$$

### 2.6. Measuring Diagonal Color Constancy Effectiveness

Effectiveness of sharpening matrices has usually been evaluated by least-squares as follows. Let us denote an observed color by ${\underline{\rho }}_{r}^{E}$ (Equation (5)) where *E* is the illuminant and *r* the reflectance for the observation. Then, if we select a canonical reflectance *s* (usually an achromatic reflectance), we can compute for each illuminant the ratio between any reflectance and the white reflectance as follows.

(43) $${\underline{d}}_{r,s}^{E}=T^{-1}{\left[\text{diag}(T{\underline{\rho }}_{s}^{E})\right]}^{-1}T{\underline{\rho }}_{r}^{E}$$

where ${\underline{d}}_{r,s}^{E}$ is a vector of dimension 3,

Let us note that if the transformation *T* perfectly accomplishes diagonal color constancy, the value ${\underline{d}}_{r,s}^{E}$ is independent from the illuminant. Therefore, measuring the disparity of this ratio depending on the illuminant should tell us the effectiveness of a method.

Mathematically, if we select a canonical illuminant *E^c^*, we can denote the error of the sharpening matrix by

(44) $$\text{Error}=100\times \frac{\left\|{\underline{d}}_{r,s}^{E^{c}}-{\underline{d}}_{r,s}^{E}\right\|}{\left\|{\underline{d}}_{r,s}^{E^{c}}\right\|}$$

This formula has been widely used to compare spectral sharpening methods and was already included in Finlayson *et al.* [15] work. By using this formula two methods outperform the rest. First, the Measurement Tensor method as shown in [29]. This method has an inherent advantage because both the method and the measure are based on least-squares. Therefore, we deal with a least square minimization-least square evaluation paradigm. The second method that excels is Spherical Sampling due to its capability to minimize any measure. Spherical Sampling presents a further advantage since it avoids local minima.

The formula presented in Equation (44) is good for a first inspection on how the methods work with simple diagonal color constancy. But, recently, further applications of sharpened sensors have been found (see next section) where this measure is no longer appropriate.

## 3. Beyond Diagonal Color Constancy

**Figure 5.**
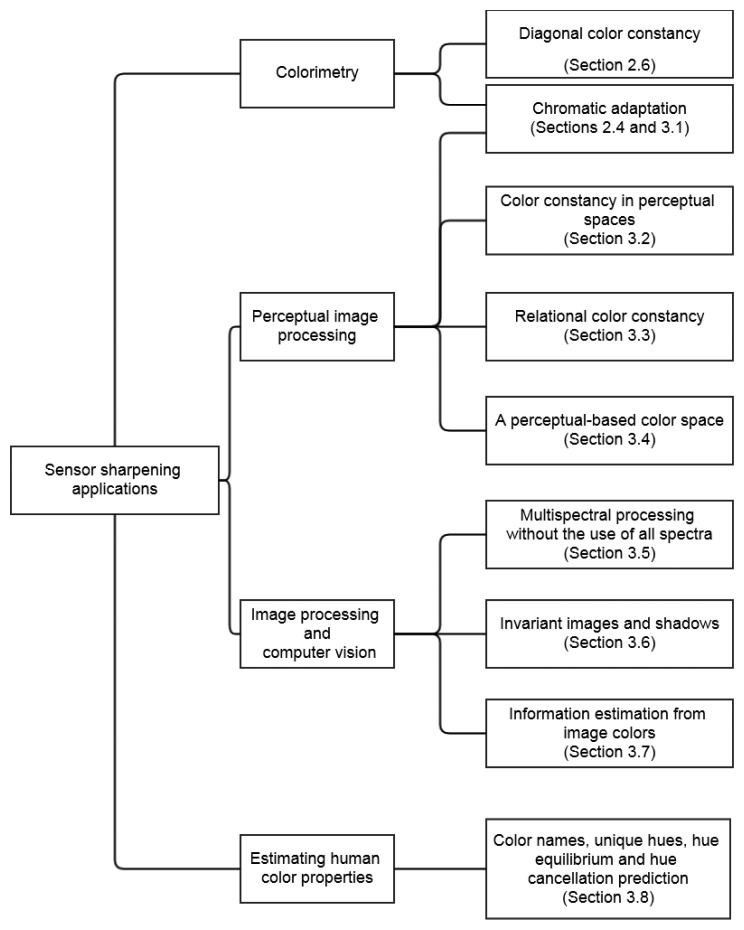
Hierarchy of sensor sharpening applications grouped by research field. Each application is linked to a section in this paper.

The original aim of spectral sharpening was to achieve diagonal color constancy. Over the years, spectral sharpening has proven beneficial for a number of purposes, some far removed from the original aim. In this section we review some of these new applications. They are presented graphically in Figure 5 where they are listed in terms of their research field and linked to a particular subsection of this paper.

### 3.1. Chromatic Adaptation

Section 2.4 shows that chromatic adaptation transforms can be understood as spectral sharpening, therefore it is a straightforward idea to use spectral sharpening techniques for handling corresponding colors data and chromatic adaptation.

Finlayson and Drew [42] showed that the Bradford transform can be obtained through spectral sharpening with a careful selection of intervals. Later on, Finlayson and Süsstrunk in [43] defined a chromatic adaptation transform following a technique very similar to the data-based sharpening considering the preservation of the white point. Ciurea and Funt [44] used the same algorithm but applied it to spectral quantities instead of tristimulus values. Finally, Finlayson and Süsstrunk [31] used the spherical sampling technique to derive a set of chromatic adaptation transforms that were equivalent in terms of the error committed to the colorimetrically obtained.

### 3.2. Color Constancy in Perceptual Spaces

Human perception is not linear but colorimetric spaces are. In other words, when we work in RGB or XYZ spaces, a Euclidean distance *d* will be perceived differently depending on the region of the color space the points are located in.

To overcome this issue CIE proposed the CIELab and CieLuv color spaces [45]. Later on, Finlayson *et al.* [41] defined a new color constancy error measure regarding differences in the CIELab perceptual space. From Equation (5), let us call *ρ̱^D^*^65^ the XYZ color value of a particular patch under the *D*65 illuminant and *ρ̱^e^* the value of the same patch under a different illuminant *e*. We know we can find an approximation of the value under the *D*65 illuminant by ${\underline{\hat{\rho }}}^{D65}=T^{-1}\cdot D\cdot T\cdot {\underline{\rho }}^{e}$. The basic idea is to convert both *ρ̱^D^*^65^ and ${\underline{\hat{\rho }}}^{D65}$ values to CIELab and to minimize the measure Δ*_ϵ_*, that is, the euclidean distance between the two points. This measure is considered to be perceptual. Formally,

(45) $$\Delta_{\epsilon }(T)=\left\|\text{Lab}({\underline{\rho }}^{D65})-\text{Lab}({\underline{\hat{\rho }}}^{D65})\right\|=\left\|\text{Lab}({\underline{\rho }}^{D65})-\text{Lab}(T^{-1}\cdot D\cdot T\cdot {\underline{\rho }}^{e})\right\|$$

The matrix *T* minimizing this equation for a set of reflectances and illuminants is defined as the best matrix regarding perceptual color constancy. It is found using the spherical sampling technique [41].

### 3.3. Relational Color Constancy

Foster and co-authors [46,47] defined color constancy as a ratio-based phenomena, not pixel-based. This view, called relational color constancy, assumes that the colors in a scene have a fixed relation between each other. Relational color constancy is also related to Retinex [48]. On the other hand, from the computer vision side, color ratios have proven useful for dealing with some particular problems such as object recognition [49] and image indexing [50].

Finlayson *et al.* [41] defined a color ratio stability measure that works in each sensor individually. They defined a vector *ḇ* (*m*-by-1) containing the colors for a set of *m* reflectances under the canonical illuminant viewed under a particular sensor, that is $\underline{b}=[\zeta_{1},\cdots ,\zeta_{m}]=[{\left(T{\underline{\rho }}_{1}^{c}\right)}_{i},\cdots ,{\left(T{\underline{\rho }}_{m}^{c}\right)}_{i}]$, where $T{\underline{\rho }}_{m}^{c}$ is the response of the sensors for reflectance *m* and canonical illuminant *c*, and subscript *i* denotes the sensor selected. They defined the vector of color ratios *a̱^c^*

(46) $$\begin{array}{lll} {\underline{a}}^{c}\left[\frac{\zeta_{i}}{\zeta_{j}};\frac{\zeta_{i}}{\zeta_{j+1}};\cdots \right]; & \zeta_{i},\zeta_{j}\in \underline{b} & \zeta_{i}\neq \zeta_{j} \end{array}$$

They considered a second vector of ratios for the same reflectances under a different illuminant *a̱^e^*. In this case, the total ratio error is defined by

(47) $$\epsilon (T)=\frac{1}{n}\sum_{e=1}^{n}\frac{\left\|\underline{a^{c}}-{\underline{a}}^{e}\right\|}{\left\|\underline{a^{c}}\right\|}$$

This error is minimized by the spherical sampling method [41].

### 3.4. A Perceptual-Based Color Space for Image Segmentation and Poisson Editing

Chong *et al.* [51] defined a perception-based color space with two main goals: (1) be linear correlated with perceived distances, that is, distances in this space might correlate with perceived ones, and (2) color displacements in this space should be robust to spectral changes in illumination, that is, if we re-illuminate two colors by the same light, the difference between them might stay equal. To obtain a space with these characteristics the authors showed that one further assumption is needed. This assumption states that diagonal color constancy must be well modeled in the space.

Therefore, the definition of the color space parametrization *F* given a point *x̱* in XYZ coordinates is

(48) $$F(\underline{x})=A(\hat{\ln}(B\underline{x}))$$

where *B* is coding a change in a color basis by converting original sensors into ones where diagonal color constancy is well characterized, *ln̂* is the natural logarithm and *A* is used to match perceptual distances. To obtain *B* authors used the measurement tensor method [29].

The authors showed the advantages of this new space in two common image processing tasks: image segmentation and Poisson editing [52].

### 3.5. Multispectral Processing Without Spectra

Drew and Finlayson [53] showed that spectral sharpening was useful to simplify the cost of calculating the modeling of the interaction of light and reflectance. Reducing this cost is important for problems such as ray-tracing. For this application they applied spectral sharpening in more than the usual 3 dimensions (red, green, and blue).

First, they defined a set of color signals, from which they obtained a n-dimensional (*n* = 5, 6, 7) basis *B*(λ) (via SVD). They sharpened this basis to improve the discernability of its information obtaining a new basis *B̂*(λ) = *TB*(λ), where *T* is a *n* × *n* sharpening matrix obtaining using the method *L*_2_ − *L*_2_ sensor-based with positivity. Then, any light or reflectance can be expressed as a coefficient vector in this last basis

(49) $$R(\lambda )=\sum_{i=1}^{N}{\hat{b}}_{i}{\hat{B}}_{i}(\lambda )$$

Drew and Finlayson showed that computing the modeling of light and reflectance using these coefficient vectors and then reconstructing back the full color signal needs less computations and that the error committed is very small.

Later on, Finlayson *et al.* [41] showed that even smaller errors are obtained by the use of spherical sampling in terms of the Δ*_ϵ_* measure between the *Lab* values of the real and the reconstructed signal.

### 3.6. Obtaining an Invariant Image and Its Application to Shadows

Finlayson *et al.* [54] theoretically proved that it is possible to obtain a 1-dimensional representation of reflectances independent from the illuminant if one supposes a narrow-band camera. From this 1-dimension representation they obtained the invariant image, where the RGB value of the pixel is substituted by its illuminant-independent representation. In the same work they also showed that the illuminant independent representation was useful in real cameras (the narrower the camera sensors, the better the results). Following this last point, Drew *et al.* [55] proved that when using spectral sharpening sensors, the invariant image was better than with the original ones.

Finlayson *et al.* [56], later used the invariant image to remove shadows from images. Drew *et al.* [57] recently showed that when using spectral sharpening sensors, the results were improved, although this last algorithm requires user interaction.

### 3.7. Estimating the Information from Image Colors

Recently, Marin-Franch and Foster [58] presented a method to evaluate the amount of information that can be estimated from the image colors captured by a camera under different illuminations. To this end, they applied different statistics to the color images. In their work they explained that when dealing with spectrally sharpened sensors the amount of information that can be extracted is higher than with the original ones.

### 3.8. Color Names, Unique Hues, Hue Cancellation and Hue Equilibrium

Philipona and O'Regan [59] showed the possibility of extracting a surface reflectance descriptor using only the information reaching our eye. To start with, they defined *&upsilon;^s^* as the accessible information about the reflected light for a given surface *s* and *u* the accessible information about the incident illuminant

(50) $$\begin{array}{ll} \upsilon_{i}^{s}=\int_{\omega }Q_{i}(\lambda )E(\lambda )R(\lambda )d\lambda , & i=1,2,3 \end{array}$$

(51) $$\begin{array}{ll} u=\int_{\omega }Q_{i}(\lambda )E(\lambda )d\lambda , & i=1,2,3 \end{array}$$

where *R*(λ), *E*(λ) are the same as in Equation (5) and *Q_i_*(λ) is the absorption of photopigments presented in the L,M and S photoreceptors.

They repeated this procedure for *N* different lights. They arranged the *N* response vectors for the surface in a 3 × *N* matrix *V^s^* and for the lights in a 3 x *N* matrix *U*. They related these two matrices by finding the best 3 × 3 matrix transform *A^s^* such that

(52) $$V^{s}≃A^{s}\cdot U$$

where the superscript *s* denotes dependence on the surface. They solved for the matrix *A^s^* by linear regression. Finally, they considered the eigenvalue/eigenvector decomposition of *A^s^* reaching to

(53) $$V^{s}≃U^{s}V^{s}{\left(U^{s}\right)}^{-1}\cdot U$$

where 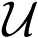
^*s*^ and 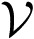
^*s*^ are the 3 ×x 3 matrices of the eigenvectors and eigenvalues respectively.

Philipona and O'Regan selected the eigenvalues 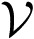
^*s*^ as the surface descriptor, from where they were able to precisely predict color naming, unique hues, hue equilibrium and hue cancellation data.

Building upon Philipona and O'Regan's model, Vazquez-Corral *et al.* [60] demonstrated that it is possible to find a unique transformation *T* for all the surfaces such that the Philipona and O'Regan model can be expressed as

(54) $$\upsilon^{s}≃TV^{s}T^{-1}\cdot u$$

This transformation *T* was computed by spherical sampling. The work of Vazquez-Corral *et al.* shows that the surface reflectance descriptor is equivalent to a Land color designator [6] computed in the space spanned by the sharp sensors.

## 4. Conclusion

Sensor sharpening was developed 20 years ago to achieve computational color constancy using a diagonal model. Over this period, spectral sharpening has proven important for solving other problems unrelated to its original aim.

In this paper, we have explained some of the differences between human and computational color constancy: human color constancy relies on the perception of the colors while computational color constancy relies on the absolute color values of the objects viewed under a canonical illuminant.

We have reviewed different methods used to obtain spectrally sharpened sensors, dividing them into perfect sharpening, sensor-based sharpening, sharpening with data, spherical sharpening, and chromatic adaptation transforms.

We have also described different research lines where sharpened sensors have proven useful: chromatic adaptation, color constancy in perceptual spaces, relational color constancy, perceptual-based definition of color spaces, multispectral processing without the use of all the spectra, shadow removal, extraction of information from an image, estimation of the color names and unique hues presented in the human visual system, and estimation of the hue cancellation and hue equilibrium phenomena.

## Notes

**Author Contributions**: Both authors contributed equally to this work.
